# Breast Metastatic Localization of Signet-Ring Cell Gastric Carcinoma

**DOI:** 10.5402/2011/426150

**Published:** 2010-10-27

**Authors:** C. Parrell Soler, A. Palacios Marqués, L. Saco López, R. Bermejo De las Heras, S. Pertusa Martínez

**Affiliations:** Servicio de Obstetricia y Ginecología, Hospital Marina Baixa, Avenida Alcalde En Jaume Botella Major 7, 03570 Villajoyosa (Alicante), Spain

## Abstract

Metastatic tumors in the breast are quite rare and constitute 0,5 to 6% of all breast malignancies. They often occur in a polymetastatic context. Gastrointestinal lesions rarely metastasize to the breast. The first case of a metastasis deposit to the breast and ovary from gastric signet-ring cell carcinoma was reported in the literature in 1999. Since this report, only 5 cases have been reported. We present a case report of a 37-year-old woman who complained of a lump in the left breast. Two months earlier, the woman underwent a subtotal gastrectomy and a total hysterectomy with double anexectomy, which histologically was diagnosed of gastric signet-ring carcinoma, disseminated with Krukenberg's tumor. In those days, the patient was following a chemotherapy treatment. A core needle biopsy of the lesion in left breast revealed cells with signet-ring features, with probably gastric origin.

## 1. Case Report

A 37-year-old woman complained of a lump in the left breast. Two months earlier, the woman underwent a subtotal gastrectomy and a total hysterectomy with double anexectomy. The pathologic findings were diagnostic of gastric signet-ring carcinoma, disseminated with Krukenberg's tumor. In those days, the patient was following a chemotherapy treatment.

**Figure 1:**
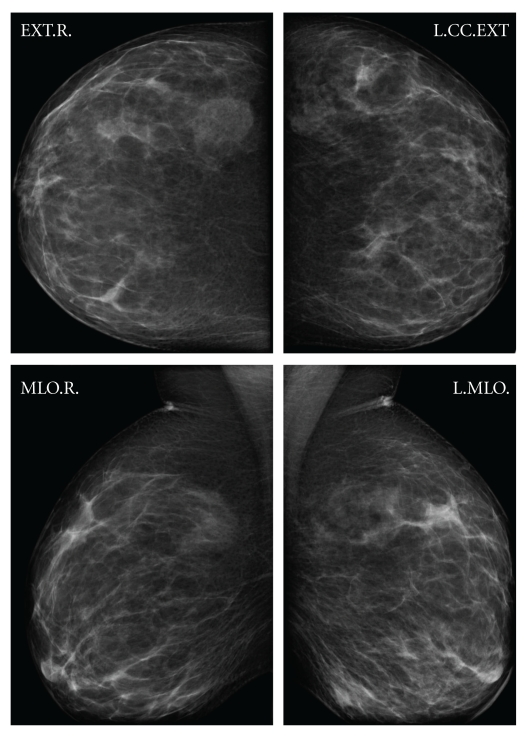
The mammography revealed asymmetric density in the lower inner quadrant of the left breast.

The examination showed an irregular area of induration, of 4 cm approximately, in lower inner quadrant of the left breast Figure 1, without evidence of lymphadenopathy. The mammography revealed asymmetric density in the lower inner quadrant of the left breast, without evidence of axillary or supraclavicular lymphadenopathy. The ultrasound exam showed a solid 1.5 cm node suspicious of malignancy. 

**Figure 2:**
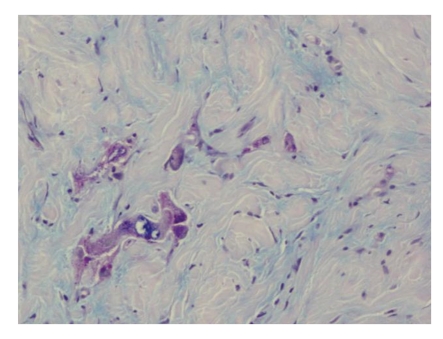
Marked fibrosis with the presence of loose cells of big size and wide cytoplasm, positive with PAS technique. The morphology is comparable to the gastric carcinoma diagnosed two months earlier.

A core needle biopsy of the lesion in the left breast revealed cells with signet-ring features, with probably gastric origin Figure 2. 

The histopathological features were CK 7 positive and C-ERB-B2, CK 20, ER, and PR negative.

The breast metastasis responded well to chemotherapy, and the size was reduced in posterior controls, but cancer progressed with peritoneal dissemination that required to be removed. The progression was finally irreversible, and the woman needed palliative care. 

The patient died 7 months after the breast metastasis was diagnosed and 9 months after the gastric tumor.

## 2. Discussion

Metastatic tumors in the breast are quite rare and constitute 0,5% to 6% of all breast malignancies. They often occur in a polymetastatic context [1]. Approximately 300 cases of breast metastases from extramammary sites have been reported [2]. The most frequent primitive tumors are lymphoma, leukaemia, and malignant melanoma [1, 3].

Gastrointestinal lesions rarely metastasize to the breast [3]. The first case of a metastasis deposit in the breast and ovary from gastric signet-ring cell carcinoma was reported in the literature in 1999 [4].

Since this report, only 5 cases have been reported [5]. Selective invasion of hormone-dependent organs seems quite intriguing, especially in premenopausal women. Increased blood supply of the breast has been proposed as the mechanism for the increased incidence of breast metastasis in premenopausal women. On the other hand, gastric cancer seems to have a more aggressive biologic behaviour in younger age groups, where hormonal factors are implicated [2].

The average age of patients at the time of presentation of breast metastases is 47 years [2].

On mammography, the metastatic lesions may appear as benign lesions, well circumscribed masses with no microcalcifications. The metastatic lesions are usually palpable and most often located in the upper outer quadrant of the left breast. Breast involvement is bilateral in 25% of the cases, and there is concomitant axillary lymph node enlargement in up to 15%. The occurrence of multiple tumor nodules is unusual. We must bear in mind that in up to 25% of patients the primary lesion has not been diagnosed yet and the palpable mass could be the first sign of an unknown disease [2, 5].

In cases of breast inflammation or lumps, biopsy should be performed, even in the presence of an extramammary neoplasm [5].

Histopathologic examination of the lesion may be usefull by distinguishing a primary breast cancer from a metastatic gastrointestinal tumor. The metastases from stomach adenocarcinomas are usually positive for CEA and cytokeratin 7 and 20 (CK) and negative for estrogen receptor (ER) and progesterone receptor (PR), as well as for C-ERB-B2 (in up to 20% it can be positive). Thus, the combination of CK 20 and CEA positive staining in conjunction with negative ER staining strongly supports a diagnosis consistent with gastrointestinal primary adenocarcinoma rather than a primary breast carcinoma [2, 3, 5, 6].

The histopathological features in our case were CK 7 positive and C-ERB-B2, CK 20, ER, and PR negative.

Wide local resection, radiation, and axillary node dissection are far from being on appropriate therapy for metastatic gastrointestinal cancer to the breast, which is associated with a poor prognosis (approximately 80% of patients die within 1 year of diagnosis) [3, 7].

It seems that the tumoral resection of primary lesion and local tumoral extension improves the survival benefits of these patients, specially when an optimal cytoreductive operation is performed [8, 9].

Our patient presented gastric signet-ring carcinoma, disseminated with Krukenberg's tumor. All these lesions were removed. When the breast metastasis was diagnosed, the patient was treated with chemotherapy, and it was decided to keep the same treatment. The breast metastasis responded well to chemotherapy, and its size was reduced in subsequent monitoring, but cancer progressed with peritoneal dissemination that was required to be removed. The progression was finally irreversible, and the woman needed palliative care.

Breast metastases with gastrointestinal primary lesion have a poor prognosis, specially those of gastric origin [7, 8, 10, 11].

Sato et al. reported two cases of breast metastasis from gastric malignant disease and marked that breast metastasis is a sign of poor prognosis of the primary malignant disease. The possibility of breast metastasis should be considered in appropriate patients to preclude unnecessary major surgery [7].

Other report of four cases of breast metastases from extramammary malignancies showed that the development of breast metastases indicates the lethal nature of blood-borne metastases in the natural evolution of a primary tumor [9].

Cheong et al. [10] studied the median survival time of patients after gastric resection and metastasectomy. The median survival time in the resection group was 17 months, which was significantly longer than that in the nonresection group, with a median survival duration of all the patients of 9 months.

Krukenberg's tumors of gastric origin have a poor prognosis [10, 11]. A study of Kim et al. with 34 female patients estimated a median survival period of 7,7 months after diagnosis. Median survival periods according to the extent of metastasis were 10,9 months for patients with disease confined to the ovaries, 13,1 months for patients with disease confined to the pelvis, 7,5 months for patients with intra-abdominal disease, and 3,6 months for patients with disease spread outside the abdomen and pelvis. It seems that the absence of residual disease after treatment and limited disease extent were favorable prognosis factors of metachronous Krukenberg's tumors of stomach origin [11].

Jiang et al. investigated survival impacts of metastasectomy in woman with Krukenberg's tumors of the ovary and survival benefits in different origins. There was a significant difference in survival between patients with metastatic disease confined to the ovaries and those with extensive metastases, with an estimated median survival of 30, 7, and 10 months, respectively [8].

Our patient died 7 months after breast metastasis diagnosis and 9 months after primary disease diagnosis.
